# Vaccine traceability: Key learnings from the supply chain initiative by manufacturers from emerging countries

**DOI:** 10.1016/j.jvacx.2023.100366

**Published:** 2023-08-04

**Authors:** Sonia Pagliusi, Yvette Madrid, Yudha Bramanti, Taufik Wilmansyah, Huilin Yu, Analia Acebal, Komarapuram R. Krishnamurthy, Venkatapathi Raju Pinnamaraju, Padmakar Jadhav, Rachel Park, Lingjiang Yang

**Affiliations:** a DCVMN International, Route de Crassier 7, 1262 Eysins-Nyon, Switzerland; b Madrid Consulting, CH-6330 Cham, Switzerland; c PT BioFarma, Jl. Pasteur No. 28, Kota Bandung, Jawa Barat 40161, Indonesia; d PT BioFarma, Jl. Pasteur No. 28, Bandung, Jawa Barat 40161, Indonesia; e Xiamen Innovax Biotech CO., LTD., #50 Shanbianhong East Road, Haicang District, Xiamen, Fujian, China; f Sinergium Biotech, Ruta Panamericana KM 38,7, Garin, CP1619 Buenos Aires, Argentina; g Bharat Biotech, Genome Valley Shameerpet, Hyderabad 500078, Telangana, India; h Vaccine Division, Biological E Limited, Shameerpet, Hyderabad, India; i EuBiologics, 8F, Seongdo Building, 207, Dosan-daero, Sinsa-dong, Gangnam-gu, Seoul, South Korea; j ChengDu Institute of Biological Products Co. Ltd, 379#, 3rd Section, Jinhua Road, Jinjiang District, Chengdu 610023, China

**Keywords:** Supply chain, Traceability, Packaging technology, Barcode, Serialisation, Counterfeit, Safety monitoring

## Abstract

The use of global standards, and the placement of barcodes and data matrix codes on vaccine labels and other levels of packaging are crucial elements for the traceability of finished vaccine products. Vaccine manufacturers are committed to improving health through their products, as vaccine production offers opportunities that can be leveraged to benefit immunization systems. In 2019 the Developing Countries Vaccine Manufacturers Network (DCVMN) created the Supply Chain Initiative aimed at prioritize and explore traceability opportunities; concomitantly procurement agencies announced traceability requirements for vaccine global supply. Vaccine traceability brings benefits including supply chain reliability and safety through enhanced product movement visibility, and a reduction of falsified and expired vaccines circulating in the supply chain. DCVMN has coordinated the development and implementation of global traceability standards, at both primary and secondary vaccine packaging levels, to encourage and enable sharing these experiences. Six pilot studies in four different countries showed successful implementation, and constituted part of larger vaccine traceability work within the respective organizations. The main findings from these pilot studies indicated that stepwise approaches to the adoption of traceability standards allowed vaccine manufacturers to learn by doing, initially with lower risk, and to spread their investments over time. Because the value of traceability is in its scale of adoption and the use of the data, it remains important for all stakeholders to engage in and prioritize the journey of vaccine traceability, but also to suitably manage the financial risks. The DCVMN Supply Chain Initiative has demonstrated that its members are committed to driving supply system changes that benefit immunization, while recognizing that supply chain traceability is part of a larger healthcare ecosystem and should be adopted by countries and immunization programmes as well.

## Introduction and background information

Traceability for medical products can be defined as the ability to track and trace a product from a determined starting point, e.g. from the manufacturing facility, to some determined endpoint in the medical supply chain. One implication of traceability is the ability to be able to uniquely identify products movement. In addition, some means to record the location and history of the product origin and movement towards end users are also usually necessary. Considering this need, the World Health Organization (WHO) has recently developed a policy paper on traceability of medical products [1].

**Fig. 1:**
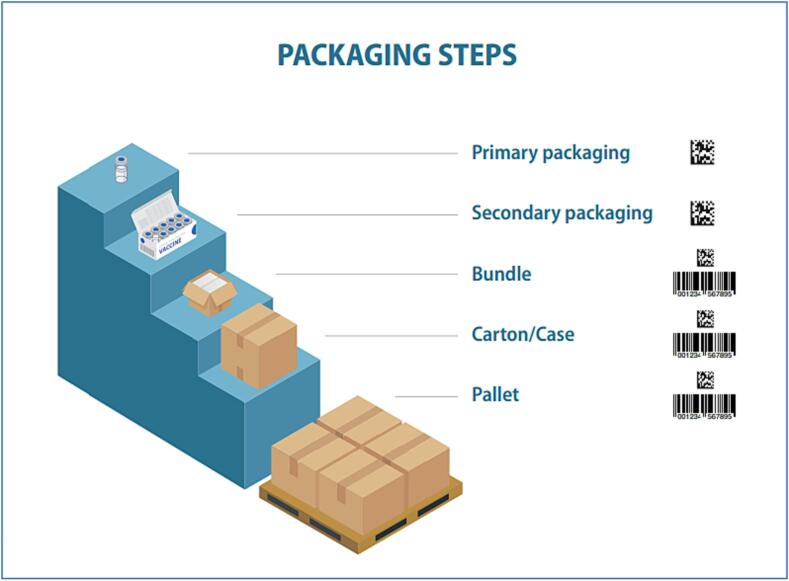
Packaging levels and corresponding traceability technology tools. Implementing serialization technology encompasses challenges beyond compliance at all stages of the packaging process due to the numerous steps and levels of packaging involved, from primary vials and secondary packaging carrying 2D data matrix, to tertiary boxes, cartons to pallets for shipment, carrying also bar code labels and GITN.

Global standards for traceability provide a key tool in assuring robust and accurate product identification and movement, and also have established processes for updating involved users. The benefits of using these global standards include the interoperability across supply chain stakeholders as well as within safety monitoring systems. GS1 global standards are widely used in healthcare [2], with the data matrix or barcode used on packaging below the pallet level to carry information (Fig. 1). GS1 established the Global Trade Item Numbers (GTINs) as the globally unique code identifiers for each product item that incorporate the company prefix, as to a central register, an item reference number, as well as digits indicating the level of packaging. Serial numbers can be assigned to provide the most granular unique identification of each unit of specific products, and these are used together with GTINs, not as replacements for them. Serialization (and aggregation) of packaging from primary levels upwards is most useful to combat falsified vaccines [3], [4].

Vaccine traceability is a key feature in the implementation of immunization information systems, helping to plan and manage immunization activities and resources, ensuring that adequate quantities of vaccines are always available to meet demand. It can streamline vaccine and ordering inventory, supply chain management, and safety monitoring, all directly of concern to manufacturers [5]. In addition, vaccine procurement tenders issued by UNICEF and based on Gavi financing, required to have GS1 standard barcoding on secondary packaging by latest 31 December 2021 [6]. The number of countries which have already implemented and/or are exploring the implementation of traceability systems continues to grow [7].

Sharing an understanding of how traceability systems work and collecting real-world experiences in their implementation is increasingly important. Therefore, and inspired by discussions held at a training workshop on supply chain traceability, held in January 2019 in Shenzhen [8], the Supply Chain Initiative of the DCVMN International was established. Specific needs were identified by a voluntary survey, designed by an independent expert, covering eight supply focus areas, and applied to DCVMN members, followed by a consultation with manufacturers, in June 2019, where the survey results and areas of common interest were prioritized [9]. This process identified a roadmap for further work to strengthening vaccine supply chain and traceability as a priority [5].

During another Supply Chain Working Group meeting, held in November 2019, PT BioFarma presented its work on vaccines traceability, which was documented [10] and inspired a project to accelerate the implementation of GS1 2D data matrix barcodes on secondary or primary packaging, through supporting pilot studies, coordinated through a purposely created Traceability Pilot Study Consortium [11]. The COVID-19 pandemic and the key role of vaccines, and their distribution, have further highlighted supply chain risks, such as counterfeiting [12] and optimization that can benefit from traceability systems. The work which ensued was moved forward through a series of working group meetings held from 2020 through 2022 [11], where manufacturers voluntarily shared non-confidential information and their perspectives of both feasibility and the constraints. The outcomes of six traceability pilot studies are provided in this report.

## Methodology

In November 2020, the DCVMN secretariat issued a call for Expression of Interest to all its member companies, for traceability pilot studies: to voluntarily implement GS1 traceability 2D codes on primary or secondary vaccine packaging. Following recommendations of an independent technical review group of experts, who met on 08 February 2021, six projects were selected, with four of these receiving donor financing, through DCVMN secretariat, for study design and on-site expert training only; equipment, supplies and staff costs were self-financed by manufacturers. As the majority of the pilot studies were implemented during the COVID-19 pandemic, there were delays of materials' delivery and travel of trainers that impacted several of the projects.

Further, participants within this group also developed an interest to explore how data matrix, barcodes and other traceability tools, including QR codes, could be used to improve traceability of raw materials and supplies within warehouse management systems (WMS). From this interest, one pilot study was implemented in 2022, with support from donor financing through DCVMN, and learnings from this pilot are also included in the report below, under point 3.2.

## Results

In total, six DCVMN member companies, from four different countries, voluntarily participated in the development and implementation of different vaccine traceability pilot studies, through the Consortium, with the majority of pilot studies on packaging being implemented from June 2021 to June 2022.

The goal of the initiative was to encourage implementation and demonstrate feasibility within a reasonable time window. Results reported here below aimed to share the implementation experiences with the broader vaccine manufacturers’ community from emerging markets. The outcomes of the six pilot projects were assessed qualitatively, rather than quantitatively, in order to respect confidentiality and avoid direct comparison of performance between participating manufacturers. Also due to the very small number of different pilot studies, from different countries on different products, statistical analysis was not sought.

**Fig. 2:**
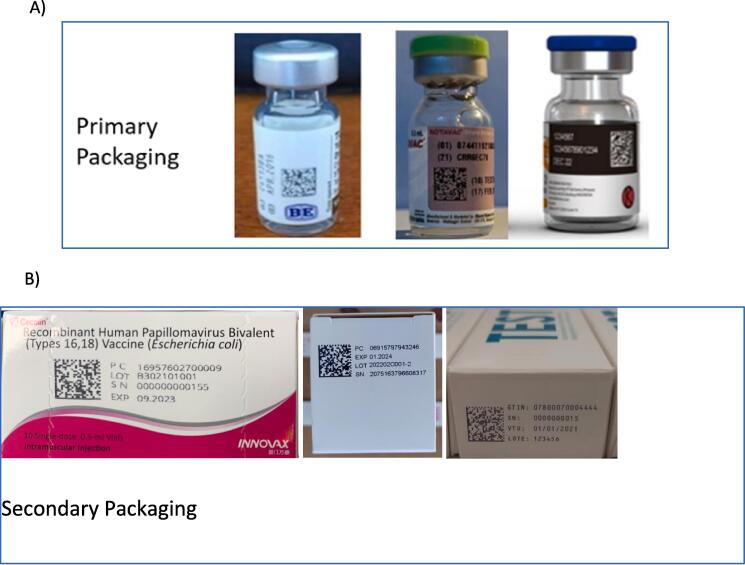
Pictures of the six primary and secondary packaging labels only. A) Pictures of the primary packaging displaying the 2D data matrix codes on the labels of the primary package, vials containing the vaccines (COVID-19 vaccine from Biological E, India; Rotavirus vaccine from Bharat Biotech, India; COVID-19 vaccine from PT BioFarma, Indonesia); B) pictures the secondary packaging, i.e. boxes containing the vaccine vials (HPV vaccine from Innovax, China; Japanese Encephalitis vaccine from ChengDu Institute, China; influenza vaccine from Sinergum Biotech, Argentina).

Three pilot studies involved traceability through serialization (data matrix) on secondary and higher levels of packaging, and all participants purchased and upgraded equipment (i.e. cameras, printers, control panels), modified their packaging lines for cartons and cases, and installed or modified software. However, the key challenge areas for implementation varied (see Table 1 and text below). For one manufacturer, software issues proved the most challenging; for another manufacturer, process adjustments and training; and for the third manufacturer, hardware installation, validation and qualification were most challenging. Implementation times also varied substantially, from six months to twenty months with all three efforts suffering from COVID-19 delays. An overview of the scope, timeframe, some technical details and challenges of these pilot studies is provided in Table 1. Despite challenges, all three secondary-level pilots fully achieved their objectives (Fig. 2).

**Table 1:** Summary of six pilot studies focused on primary and secondary vaccine packaging.

Level of packaging for labelling implementation	Company	Previous experience with GS1 tools	Market of Focus for the pilot study	Study part of a larger project	Time to implement (months)	Delay due to COVID-19 pandemic (months)	Use of serialization tools	Key challenges (excluding pandemic issues)
PRIMARY PACKAGING	Manufacturer 1	Yes, secondary/tertiary level with serialization	UNICEF	Yes. Serves as an internal pilot for expanding GS1 data matrix to primary packaging.	3	6	Yes	Artwork/Space Limitations (for smallest vial size)
	Manufacturer 2	Yes, secondary/tertiary level with serialization	National	Yes. Serves as an internal pilot for expanding GS1 data matrix to primary packaging.	2	3	Yes	Artwork/Space Limitations (for smallest vial and small label size)
	Manufacturer 3	Yes, secondary/tertiary level with serialization	National & UNICEF	The pilot was the start of an extensive project including serialization and labeling at every level and software development for track and trace in national distribution.	12	none	Yes	Software integration due to expanded traceability scope
SECONDARY PACKAGING	Manufacturer 4	QR codes	UNICEF	No	6	2	Yes	Process Changes & Training Artwork/Space Limitations Software
	Manufacturer 5	QR codes	UNICEF	Yes	11	2	Yes	Software Process Changes & Training Regulatory
	Manufacturer 6	No	National, for private market	Plan to extend to primary packaging with full serialization due to national regulation	20	8	Yes	Hardware Software Process Changes &Training

Three manufacturers undertook pilots focused on secondary-level packaging, and another three manufacturers, that already had experience in secondary level, chose to implement on primary-level packaging. Elements that contributed to the implementation include previous experience with GS1, study part of a larger project, market focus, time of implementation and delays, use of serialization tools and specific challenges are outlined.

Three other pilot studies involved placing GS1 data matrix barcodes on primary vaccine packaging. The detailed experience of one of these, PT BioFarma, has been documented in a previous publication [10]. It is worth highlighting that of the six traceability projects, the PT BioFarma experience is the one with the broadest scope. Their pilot study served as basis for an initial phase of a large scale project and was triggered by a national response in Indonesia to falsified vaccines [13]. However, the project would include vaccines for UNICEF procurement and was modified after the pilot and during the pandemic, to also include COVID-19 vaccines. One key project objective was to label GS1 data matrix codes on both secondary and primary packaging, with appropriate serialization and aggregation. Beyond this, as PT BioFarma is a publicly owned institution, it also developed software solutions that went beyond its own needs and enabled vaccine traceability throughout the distribution channels in Indonesia national healthcare system. Efforts to facilitate the sharing of data across borders are continuing through the use of GS1 Electronic Product Code Information Services (EPCIS) [14].

Two additional manufacturers also developed pilot studies with a focus on primary vaccine packaging. These producers had the most extensive prior experience with GS1 standards, of any of the six producers that developed pilots, due to longstanding national traceability requirements on products for export which were first announced already in 2011 [15]. However, the focus until now had been on secondary packaging. For the effort on primary packaging, one manufacturer focused on rotavirus vaccines for UNICEF procurement, as this constituted a continuation of previous work, to add GS1-compliant secondary labels to these products, although the expansion to primary packaging for rotavirus exceeds the current UNICEF/WHO/Gavi requirement, while anticipated local requirements. The other manufacturer decided to focus its pilot on COVID-19 vaccines.

For all three producers, these pilot studies explored the changes to labels, hardware, software, and processes associated with placing serialized data matrix barcodes on primary packaging and assuring appropriate aggregation at higher levels of packaging. For all three producers, the pilot served to inform expanded efforts to place serialized GS1 data matrix barcodes on primary packaging. For the Indian producers, these pilots have proved timely, as requirements have recently shifted to now incorporate this primary level of packaging [15]. All the objectives of the primary packaging pilots were achieved. Additional insights into these pilots are provided in Table 2.

**Table 2:** Success-enabling factors for implementation of vaccine traceability tools.

**Success-Enabling Factors**	**Highest # found useful/ Most used/ Minority used**
Contracted support through a certified vendor/solution provider	Highest
Consortium Coordinated Meetings and Knowledge Sharing	Highest
Technical Advice from GS1	Highest
Establishment of in-house multi-disciplinary teams	Highest
Frequent interactions with regulator and/or UNICEF/WHO PQ	Most
DCVMN donor financial support	Most
Attract additional financing/investors	Minority

Factors that enabled successful implementation of the pilot studies as indicated by the manufacturers themselves, upon a post-implementation anonymous survey, conducted online. The outcomes are related to the six participating companies, and the seven most success enabling factors are listed in order of choices.

### Some factors that contributed to successful implementation of vaccine traceability tools

All the companies had some previous experience with GS1 standards on barcode labels, although one was relatively new to it and benefited from the training support of a contracted service provider as well as, certified by GS1. Additional insights can be gleaned from this group of six studies. National regulation and/or requirements from UNICEF/Gavi/WHO, or the anticipation of these, are key motivators of the traceability projects. All the studies, except for one were linked to extended work in traceability demonstrating that a step-wise approach to implementing traceability is used by manufacturers. This allows producers to learn by doing with less risk and it also allows them to space the financial and other resources over time, making the efforts and investment more manageable. Considering this together with the key role of regulatory mandates to drive progress, the results suggest that frameworks with well-thought long-term plans for traceability roll-out, that incorporate a stepwise approach, will facilitate the necessary interventions of more manufacturers.

There were similarities in the process steps of the initiatives as well as differences. The differences hinge largely on initial processes for getting started (due to varying levels of experience with GS1 standards); the degree and nature of external support provided through certified vendors; and the enhanced complexity associated with adopting serialized barcodes on primary labels or, in the case of PT BioFarma, in also developing traceability software for distribution.

All the pilot studies, save one, used contracted GS1 certified vendors to support planning and training aspects of the work. Where these were used, user requirement specifications (URS) developed by the manufacturers were used to clarify expected user requirements to the vendor. These can vary in scope.

Manufacturers, vendors, or both working in concert examined feasibility and gap analyses to clarify the needs. Items for purchase, such as cameras, hand-held scanners, printers or carton-coding machines, were specified, selected, purchased, installed, and verified. Packaging lines were upgraded, and flow was adjusted, sometimes allowing for both manual and continuous automated steps, sourced by manufacturers themselves. The artwork for labels and cartons was re-designed. Label modifications were also submitted to regulatory authorities and/or to WHO Pre-qualification as post-approval changes.

Software to verify packaging readability and ensure appropriate aggregation was upgraded. Real-time data coming from the packaging lines could be linked to enterprise resource planning systems (ERPs). Quality qualifications were conducted at appropriate times in the process. Installation qualification (IQ) ensured that equipment or software conforms to specifications and is appropriately installed. Operational qualification (OQ) ensured that each piece of installed equipment or software performs as it should. Performance qualification (PQ) ensured the entire system functions as it should. Standard operating procedures (SOPs) were modified, and staff was trained accordingly. Some factors that contributed to success were identified by an online anonymized survey conducted post-implementation (in November 2022), as summarized in Table 2. Support of certified vendor/solution provider, group coordinated meetings, GS1 technical advice, and multi-disciplinary teams were the highest factors contributing to success.

Given the large variation across pilot studies in scope, complexity, and previous experience, it is inherent that there is also a large variation in associated costs. There is little value in this context to discuss average costs. Hence, an indicative range across all these projects (by scope and complexity) is US$ 200,000 to US$ 2,000,000 dollars, not adjusted for inflation or purchasing power. These figures should be interpreted and used with care. Implementation at full scale (all vaccines, all packing lines, all markets) has different financial implications than piloted changes for one vaccine/one line.

It is also worth noting that four of these pilots received some small financial support through DCVMN. These funds were used in ways to enhance the success of the projects and the speed of their implementation, such as contracting external expert certified service support. It is possible that this financing catalyzed the implementation projects in cases where there was no specific regulatory requirement to meet, such as primary packaging mandatory 2D codes (two primary packaging pilots), and there was a manufacturer with experience interested to expand traceability to additional products. The costs are important, and merit planned investment, as for many producers, costs may be prohibitive, even if adoption follows a stepwise approach.

### Seeking end-to-end traceability through warehouse management systems

**Fig. 3:**
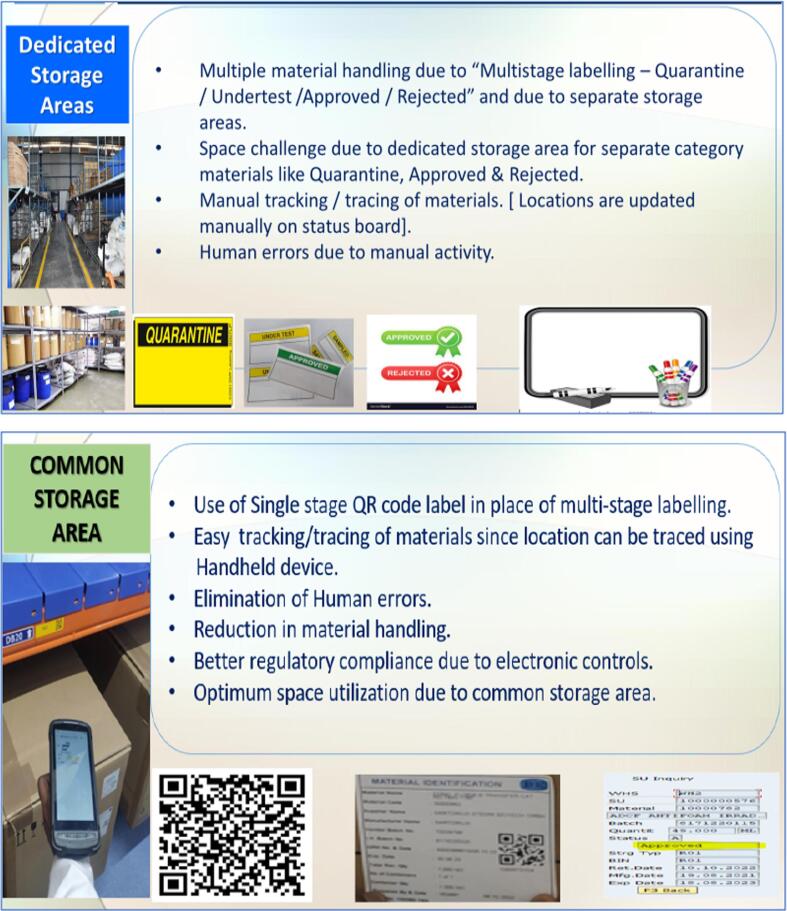
Results of Warehouse Management System upgrade pilot study. Upper panel illustrates the challenges of warehouse management system implementation towards higher digital performing settings, including mannual multilabelling activity. Lower pannel illustrates the benefits of integrating information on status and bin level of materials/supplies segregation using QR code to check material characteristics, after implementation of digitalized information system for warehouse management.

The vaccines traceability Consortium also expressed interest in exploring Warehouse Management Systems upgrade through pilot studies. A Call for Proposals to DCVMN members was issued in March 2022 and one project, was selected for implementation from June to October 2022, using software from SAP^1^. The purpose of this pilot was to reduce inefficiencies in the management of incoming raw materials and supplies that are used in drug substance production and packaging. Due to a lack of digital tracking, separate storage locations were required to quarantine materials, resulting in inefficient use of space (Fig. 3). Label changes indicating status (quarantine, test, approved) were also necessary. The manual tracking and tracing of material within the warehouse also contributed to human error and inefficient use of time.

The steps in implementation involved the selection of a vendor, development of the software application, finalization of the application including training and cutover strategy, and computer system validation (CSV) incorporating IQ, OQ, and PQ.

The following challenges were faced:

• the preparation, verification, and management of data while continuing to operate;
• delivery of network switches and wi-fi access points;
• user acceptance test for all business scenarios; and
• placement of QR labels on available inventory.

The project objectives were met after three months resulting in the following advantages:

• improved reliance of tracking systems;
• fewer errors due to reduced manual steps;
• improved regulatory compliance linked to digital controls;
• optimized stage due to common storage area; and
• simplified labeling resulting from the use of a single QR code.

## Discussion

These pilot studies provide some valuable learnings. Notwithstanding a diversity of objectives, experience, and challenges, all the pilot studies were successful. Packaging and labeling modifications to support finished product traceability are broadly achievable. However, manufacturing efforts strongly benefit from well-thought legislation, regulation, and requirements developed by countries and global stakeholders which see traceability as a journey with stepwise progress. Manufacturers can and do take initiative when they feel that the effort anticipates regulation and/or provides operational benefits to them, such as in warehouse management. Implementation is a learning process and manufacturers in these pilots have learned by doing, as well as through the sharing of experiences. They also have leveraged other factors, such as the establishment of multi-disciplinary in-house teams, the contracting of certified vendors, and support from GS1, to achieve success.

WHO’s vaccine pre-qualification and UNICEF’s procurement requirements were the drivers for two manufacturers (Innovax and CNBG), while other two (Bharat and Sinergium) sought to meet national/domestic traceability requirements for vaccines destined for the public or private market. In addition, the COVID-19 pandemic was a driver of implementation of primary packaging 2D labelling.

Still, there are challenges for traceability that lie ahead. Barcodes on vaccine packaging are carriers of data but do not ensure traceability in the field on their own. Traceability occurs when data are captured, shared, and used. This implies that software solutions and hardware supporting traceability in distributors and field users need to be built and purchased. Processes should be adapted to shift from manual to digital means of data capture and use. And all this should be done at a sufficient scale, within countries, across stakeholders and borders. Despite progress, making this happen in a timely fashion at scale implies prioritizing this work across more manufacturers, countries and stakeholders. There are challenges to this. For example, in sub-Saharan Africa a recent review found that the region had embraced eHealth and digital interventions, but “It is lacking in co-ordination, integration, scalability, sustainability, and equitable distribution of investments in digital health” [16]. It is promising that global immunization stakeholders, such as Bill & Melinda Gates Foundation, Gavi, the Global Fund, UNICEF, VitalWave, the World Bank and countries are now working together to develop and Implement TRVST [17]. This track-and-trace initiative has been piloted in Rwanda and Nigeria with more countries indicating an interest in its adoption [7].

Country traceability visions and regulatory frameworks serve as an important impetus for manufacturers to act. Mandates should aim for as much standardization as possible to avoid a multiplicity of requirements and labels that increase costs for manufacturers. Manufacturers should also embrace their partnership role and continue their traceability journey by expanding products and levels of packaging that support traceability. Manufacturers’ initiatives to incorporate GS1 barcode matrices on vaccine packaging labels are largely driven by existing, or expected national regulations and/or requirements placed by UNICEF/Gavi/WHO. This enables, but is only one component of, finished product traceability. The systems for tracking product movement and sharing data should also be established, although this responsibility is typically not one that manufacturers undertake. Furthermore, it is also possible to establish traceability earlier in the production process and into the warehouse management system (WMS). Here, it is the manufacturer which drives the process, primarily to digitize warehouse processes. In addition, this investment can also improve regulatory compliance and can contribute to larger end-to-end product traceability.

Some potential benefits of traceability systems, all of which are relevant to immunization systems, include:

• improved supply chain management due to near real-time information on product movement and stock levels and reduced manual error;
• reduction of falsified and/or unauthorized and/or expired products entering the supply chain or being inadvertently distributed to end users;
• increased patient safety through the ability to link individually identified products (through serialization) to patients;
• improved ability to associate products with adverse events;
• improve reaction time in the case of product recalls.

These are benefits that largely accrue to the health system and patients, although there are also important benefits for manufacturers, such as inventory control and facilitation of commercial processes of order fulfillment. Falsified products of lower quality carrying the brand labels of known manufacturers, for example, are risks for patient safety but also to a producer’s reputation. The ability to better address pharmacovigilance and product recalls benefits regulatory systems, producers and ultimately the vaccinees.

The challenges in establishing these systems have delayed implementation in some regions and areas, and include:

• need for robust and accurate product information including a standardized means of identifying products and key product attributes, such as lot numbers and expiry dates across supply chain stakeholders;
• capacity for capturing and sharing data at key distribution points along the supply chain;
• systems to securely manage and store large amounts of data;
• digital systems for sharing product and movement data across supply chain stakeholders;
• regulatory systems with resources and knowledge capable of ensuring the establishment and the appropriate implementation of these systems across stakeholders.

## Conclusion

The DCVMN Supply Chain Initiative has focused its efforts on enabling a more profound understanding of traceability, stockpiles, and packaging innovation that can be leveraged to improve health systems outcomes [5]. Supply chain improvements associated with secondary production can have a considerable positive impact on immunization objective, e.g. increasing ease of use and coverage, track-and-trace in supply chains, and immunization safety. This asymmetry that exists between where the effort is made (secondary production) and where the gain is (health systems) is important to recognize, particularly for use in pandemic settings [18]. This initiative demonstrates that it is possible and important to make use of this leverage, and that investments and risks should be shared across stakeholders. Manufacturers have a key role in supply chain improvements that are driven by secondary vaccine production, but they cannot implement these alone.

Improvements in vaccine traceability, stockpiles, and innovative packaging require systemic shifts with manufacturers working together with other stakeholders from suppliers, regulators, to purchasers and multi-lateral organizations to align their objectives and efforts. This includes:

• Prioritization, investment, and co-ordination of traceability efforts by all stakeholders; in plans and financing of the full costs and risks of vaccine stockpiling;
• Recognition that innovation adoption requires that novel technology be matched with clear signals of willingness to pay.

## Note

This report summarizes the views of an international group of professionals and reflects their opinions in a given point in time and context, and does not represent the decisions or the stated policy of any institution or corporation with which the authors are affiliated.

## Declaration of Competing Interest

The authors declare that they have no known competing financial interests or personal relationships that could have appeared to influence the work reported in this paper.

## Data Availability

Data will be made available on request.

## Footnotes

1 SAP stands for Systems Applications and Products in Data Processing. SAP, by definition, is the name of the ERP (Enterprise Resource Planning) software as well as the name of the company - SAP Software - an European multinational, founded in 1972 in Germany, by Wellenreuther, Hopp, Hector, Plattner, and Tschira.
